# Paths taken by Brazilian Nursing for the development of terminological subsets

**DOI:** 10.1590/1518-8345.3132.3270

**Published:** 2020-05-11

**Authors:** Harlon França de Menezes, Alessandra Conceição Leite Funchal Camacho, Maria Miriam Lima da Nóbrega, Patrícia dos Santos Claro Fuly, Sâmara Fontes Fernandes, Richardson Augusto Rosendo da Silva

**Affiliations:** 1 Universidade Federal Fluminense, Escola de Enfermagem Aurora de Afonso Costa, Niterói, RJ, Brazil.; 2 Hospital Pró-Cardíaco, Unidade Coronariana, Rio de Janeiro, RJ, Brazil.; 3 Universidade Federal da Paraíba, Departamento de Enfermagem em Saúde Coletiva, João Pessoa, PB, Brazil.; 4 Universidade do Estado do Rio Grande do Norte, Faculdade de Enfermagem, Natal, RN, Brazil.; 5 Universidade Federal do Rio Grande do Norte, Departamento de Enfermagem, Natal, RN, Brazil.

**Keywords:** Nursing, Classification, Terminology, Nursing Process, Nursing Research, Postgraduate Nursing Education, Enfermagem, Classificação, Terminologia, Processos de Enfermagem, Pesquisa em Enfermagem, Educação de Pós-Graduação em Enfermagem, Enfermería, Clasificación, Terminología, Procesos de Enfermería, Investigación en Enfermería, Educación de Postgrado en Enfermería

## Abstract

**Objective:**

to discuss the paths taken by Brazilian Nursing in the development of terminological subsets of the International Classification for Nursing Practice.

**Method:**

documentary research, carried out in master’s dissertations and doctoral theses, which developed terminological subsets, available at the Bank of Doctoral Theses and Master’s Dissertations of the Under-graduation Personnel Improvement Coordination. The variables were analyzed were institution, year; academic level, type of health service, methodological approach, clientele, theoretical reference, validation of terms, cross mapping, modeling of new concepts, validation of statements, method used for elaboration, term collection, finalization and dissemination.

**Results:**

124 doctoral theses and master’s dissertations were found, 91 were excluded and 33 were included, 23 (69.70%) of which were master’s dissertations, with the highest production in 2014 (n=10; 30.30%), with emphasis on the Northeast (36.36%); the ‘Primary Care’ scenario, with six studies (18.18%); and the predominant clientele was cancer patients. As for the methodological characteristics, in 96% of the studies, the quantitative approach was used; in 2%, a qualitative approach; and 2% associated the quantitative and qualitative approaches. As for the type of study, 60% were methodological and 24% descriptive-exploratory, with the Horta model being the most used (36%).

**Conclusion:**

the paths are successful, yet still permeated by weaknesses in the validations and potentialities to standardize the language.

## Resumo

**Objetivo:**

discorrer sobre os caminhos percorridos pela enfermagem brasileira no desenvolvimento de subconjuntos terminológicos da Classificação Internacional para a Prática de Enfermagem.

**Método:**

pesquisa documental, realizada nas dissertações de mestrado e teses de doutorado, que desenvolveram subconjuntos terminológicos, disponíveis no Banco de Teses e Dissertações da Coordenação de Aperfeiçoamento de Pessoal de Nível Superior. Foram analisadas as variáveis: instituição, ano, nível acadêmico, tipo de serviço de saúde, abordagem metodológica, clientela, referencial teórico, validação de termos, mapeamento cruzado, modelagem de novos conceitos, validação das afirmativas, método utilizado para elaboração, coleta de termos, finalização e divulgação.

**Resultados:**

foram encontradas 124 teses de doutorado e dissertações de mestrado, excluídas 91 e incluídas 33, sendo 23 (69,70%) dissertações de mestrado, com maior produção em 2014 (n= 10; 30,30%), com destaque para o Nordeste (36,36%); o cenário ‘Atenção Básica’, com seis estudos (18,18%) e a clientela predominante foi de pacientes oncológicos. Quanto às características metodológicas, em 96% dos estudos, empregou-se a abordagem quantitativa; em 2%, a abordagem qualitativa e 2% associaram a abordagem quantitativa e a qualitativa. Quanto ao tipo de estudo, 60% foram metodológicos e 24% descritivo-exploratórios, sendo o modelo de Horta o mais utilizado (36%).

**Conclusão:**

os caminhos são exitosos, contudo ainda permeados de fragilidades nas validações e nas potencialidades para uniformizar a linguagem.

**Objetivo:**

discurrir sobre los caminos recorridos por la Enfermería brasileña en el desarrollo de subconjuntos terminológicos de Clasificación Internacional para la Práctica de Enfermería.

**Método:**

investigación documental, realizada en tesis de magister y tesis de doctorado, que han desarrollado subconjunto terminológicos disponibles en el Banco de Tesis de Doctorado y Tesis de Magister de la Coordinación de Perfeccionamiento de Personal de Nivel Superior. Se analizaron las siguientes variables: institución, año, nivel académico, tipo de servicio de salud, enfoque metodológico, clientela, marco teórico, validación de términos, mapeo cruzado, modelación de nuevos conceptos, validación de afirmaciones, método utilizado para elaboración, recolección de términos, finalización y divulgación.

**Resultados:**

se encontraron 124 tesis de doctorado y tesis de magister, se excluyeron 91 y fueron incluidas 33, siendo 23 (69,70%) tesis de magister, con mayor producción en 2014 (n= 10; 30,30%), con prevalencia del Nordeste (36,36%); el escenario ‘Atención Primaria’, con seis estudios (18,18%) y la clientela predominante fue la correspondiente a pacientes oncológicos. En relación a las características metodológicas, en 96% de los estudios se empleó el método cuantitativo; en 2% el enfoque cualitativo y en 2% se advirtió la fusión entre los métodos cuantitativo y cualitativo. En lo atinente al tipo de estudio, 60% fueron estudios metodológicos y 24% descriptivo-exploratorios, siendo el modelo de Horta el más utilizado (36%).

**Conclusión:**

los caminos han sido exitosos, aunque todavía estén atravesados por déficits en las validaciones y en las potencialidades para estandarizar la terminología.

## Introduction

In the age of evidence-based health, nursing assumes its role in demonstrating that the care provided is associated with satisfactory patient outcomes and a high degree of quality and safety.

The use of standardized nursing terminologies and classification systems represents an important information tool to describe the elements of clinical practice in order to improve the quality of nursing records. Thus, it allows the continuity of care, consistency in written communication and increased safety for patients, since it makes it possible to provide data that demonstrate their contribution to health care, promoting changes through education, administration and research. In addition to resulting in greater visibility and recognition, which promotes more autonomy to the profession^(^ 1 ^-^ 2 ^)^.

This being, the International Council of Nurses (*Conselho Internacional deEnfermeiros*, CIE) seeks to universalize professional language aiming at agility and readiness in the definition of nursing diagnoses and interventions, as well as possibilities for dialogue at the international level, in different cultural, social and health contexts^(^ 3 ^)^.

Among these classifications, the International Classification for Nursing Practice (*Classificação Internacional para a Prática de Enfermagem, CIPE* ^®^) stands out as a technological resource that brings together, in the same classification, terms and concepts of nursing diagnoses, results and interventions, representing important information tool to describe the elements of clinical nursing practice^(^ 4 ^)^, being inserted in the Family of International Classifications of the World Health Organization (WHO), in order to expand the coverage of the practice domain nursing as an essential and complementary part of the professional health service^(^ 5 ^)^.

The CIPE^®^ is a milestone of the different classification systems for elements of professional practice, which was developed due to nurses’ aspiration for a system that would represent nursing practice worldwide, and aims to unite the existing systems into a single one to create a universal language of nursing. Therefore, encouraging its use is essential, since allows the projection of trends on the needs of patients, the provision of treatments and the use of resources and results of nursing care^(^ 6 ^-^ 8 ^)^.

Studies demonstrate that the development of terminology subsets of the CIPE^®^ is still incipient in the Brazilian reality, but promising and requires more discussions in the academic and practical context of nursing to operationalize and improve the method and, consequently, disseminate terminology in the national panorama^(^ 9 ^-^ 10 ^)^. This classification should be expanded through research, nursing education and its use in care practice^(^ 11 ^-^ 12 ^)^.

In this sense, graduation plays a fundamental role in the development of nursing science, considering that the courses have objectives that converge to a broader and deeper education, which enables the production of qualified human resources, consolidating scientific knowledge to act in professional practice^(^ 13 ^)^. Thus, this study is justified in view of the need to summarize and discuss the content of academic production on the development of terminological subsets in order to provide a relevant research source to help choose the best strategies for structuring terminology subsets of CIPE^®^, thus ensuring greater quality and reliability, as well as helping to conduct new studies.

In view of the relevance of recognizing the steps taken by the researchers and the reflexes in practice, the possibilities for contributions in this investigation occur with the aim of generating new intellectual nursing processes and validating their practice. These can be incorporated into electronic health records and used to generate nursing knowledge, in addition to demonstrating its importance as a technological resource, supporting nurses’ actions in the most diverse contexts of professional practice^(^ 14 ^)^.

For the development of the study, the following research question was established: What are the characteristics of the master’s dissertations and doctoral theses available in the catalog of the of the Under-graduation Personnel Improvement Coordination (*Coordenação deAperfeiçoamento de Pessoal de Nível Superior*, CAPES) that deal with the development terminology subsets of CIPE^®^? In this perspective, the objective was to discuss the paths taken by Brazilian Nursing in the development of terminological subsets of the International Classification for Nursing Practice (CIPE^®^).

## Method

Documentary type study^(^ 15 ^)^, descriptive, retrospective and with a quantitative approach in master’s dissertations and doctoral theses, which developed terminological subsets of the CIPECIPE^®^, without time limit, available at Catalog of Theses and Dissertations of CAPES, (http://catalogodeteses.capes.gov.br/catalogo-teses/) whose keyword was “CIPE^®^”.

Data were collected between May and July 2018. In order to decrease probable systematic errors or studies’ measurement bias due to mistakes in the interpretation of results and design, the research was initially carried out by the main author and independently reviewed by a second author, in order to guarantee the rigor of the method and the reliability of the results. It should be noted that this analysis in pairs was also performed in the categorization and description of the results.

As for the inclusion criterion, were selected master’s dissertations and doctoral theses that were part of the CAPES Theses and Dissertations Catalog, produced by nurses and that address the development of terminological subsets, with no time limit. It is noteworthy that studies that use the term ‘catalog’ were also included, as they were built from diagnoses, results and interventions.

In order to guide the data collection, a structured guide was elaborated that contained the following variables for the study: Under-graduation Institution (Instituição de Ensino Superior, IES) where the work was developed; year of publication (in which the dissertation or thesis was published in full); academic level: Professional Master (*Mestrado Profissional*, MP), Academic Master (*Mestrado Acadêmico,* MA) or Doctorate (D)); type of health service analyzed (if hospital, Basic Health Unit - *Unidade Básica de Saúde*, in Portuguese, UBS or other); full title of the study; methodological approach (quantitative or qualitative); type of study (methodological, descriptive-exploratory or documentary); studied clientele; use of nursing theory and theoretical framework; validation of terms; cross mapping; modeling version of new concepts; validation of statements; method used to create subsets; term collection; finalization and dissemination (articles, works in scientific events, chapters of books or books).

A search was carried out on the website of the Lattes Platform of the National Council of Science and Technological Development (*Conselho Nacional de Desenvolvimento Científico e Tecnológico, CNPq*) authors’ curricula selected in the CAPES Catalog in order to find the scientific production originated from the main study, that is, the master’s dissertations or doctoral theses, according to the pre-established selection criteria.

The collected data were entered into a Microsoft Excel 2010^®^, spreadsheet, according to the variables, compared and contrasted in order to understand the phenomenon and respond to the objective of the study. After their descriptive analysis, they were presented in the form of tables.

**Figure 1:**
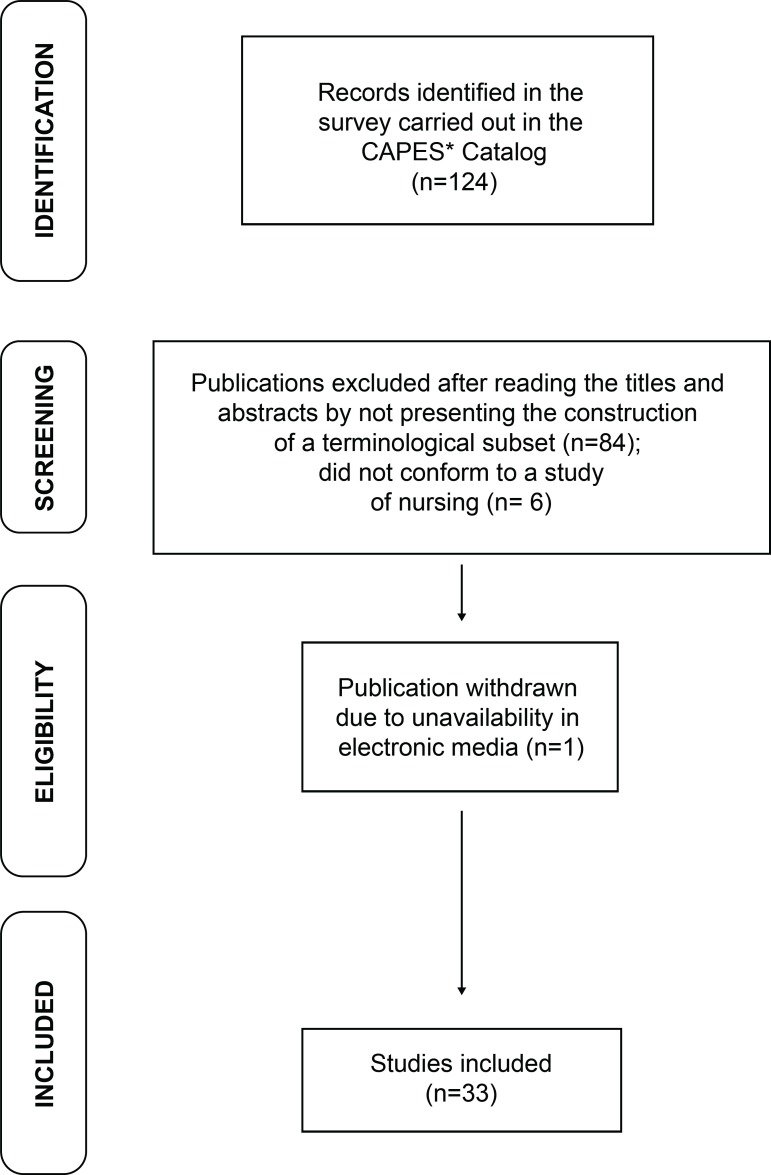
Flowchart on the methodological path to results *CAPES- Under-graduation Personnel Improvement Coordination

The flowchart presented below outlines the path of the bibliographic survey adopted by the researchers for the preparation of this research (Figure 1).

As it is a documentary study, there was no need for approval by an Ethics Committee on Research with Human Beings. However, it is noteworthy that the information selected for analysis went through peer review to certify the reliability of the results.

## Results

124 doctoral theses and master’s dissertations were identified, of which 91 were excluded because they did not present the construction of a terminological subset or did not form a nursing research, in addition to a study that is not available in full electronically. Therefore, 33 works were included in this research, being 23 master’s dissertations (69.70%) and 10 doctoral theses (30.30%).

The results indicated that the first subsets were prepared in 2009, however there was a greater number of related publications in 2014 (n=10; 30.30%), in 2017 (n=9; 27.27%) and in 2016 (n=4.12.12%). In 2013, there were 3 productions (9.09%); in 2009 and 2012, 2 (6.06%) studies each; and in 2011, 2015 and 2018, 1 study each (3.03%).

Regarding the IES, 12 were the ones that produced studies aimed at the construction of subsets, in which the Federal University of Paraíba (*Universidade Federal da Paraíba,* UFPB) stood out (n=12; 36.36%), the Fluminense Federal University (*Universidade Federal Fluminense*, UFF) and the Federal University of Espírito Santo (*Universidade Federal doEspírito Santo*, UFES), with 4 studies each (12.12%).

As for the types of health services where they were performed, primary care and the hospital environment predominated, with 6 studies (18.18%) each; in an outpatient clinic, in the oil industry, in universities and long-term care facilities for the elderly, 1 research each (3.03%). It should be noted that in 17 (51.51%) studies, health services characterized as scenarios were not identified.

The predominant clientele was cancer patients, with 6 (18%) studies, followed by the elderly, with 5 (15%). Studies with patients affected by chronic diseases, such as hypertension, diabetes and venous ulcers, as well as patients with myocardial infarction, acquired immunodeficiency syndrome, leprosy, infants, children and adolescents and patients undergoing a prostatectomy and colostomy surgical process demonstrate concern with the various human nuances. There have been studies concerning students in a university environment and workers and their association with occupational and mental health.

Regarding the use of nursing theories or models, Wanda de Aguiar Horta’s was used in 12 (36%) studies; Dorothea Orem, in 4 (12%); Virginia Henderson and Rosemarie Rizzo Parse, in 2, each (6%); Katharine Kolcaba, Callista Roy, Imogene King, Life activity model of Roper, Logan and Tierney and the Theory of Praxic Intervention in Collective Health Nursing, with 1 study each (3%). Other studies have appropriated references such as the Care Model for the Preservation of Dignity, developed by Harvey Chochinov; the Chronic Care Model, by Edward Wagner; and concepts like those of vulnerability, of José Ricardo de Carvalho Mesquita Ayres, of human development and the pathophysiological model of Congestive Heart Failure.

As for the methodological approach, in 32 (96%) studies the quantitative approach was used; 1 adopted the qualitative approach (2%) and 1 (2%) associated the quantitative and qualitative approach. As for the type of study, 20 (60%) were methodological; 8 (24%) of the descriptive-exploratory type; 2 (6%) of the descriptive type; 1 (3%) documentary, 1 transversal and 1 case study.

Table 1 shows the studies, with their corresponding titles and relates the completion of the construction of the terminological subsets, distributed according to the order of presentation at the base. Table 2 summarizes the studies described associated with the methodological path for the construction of subsets as a strategy for the collection of terms, cross-mapping with its corresponding version and the validation of statements using validation methods, with content validation being used in 21 studies (63.6%).

**Table 1:** Distribution of master's dissertations and doctoral theses extracted from the catalog

Study	IES*	Year	Academic Level	Title	Quantitative of productions
1	UFPB^†^	2009	MA^‡^	CIPE^®§^ catalog for cancer pain	Book (1), article (1), summaries at events (3)
2	UFF^||^	2012	MP^¶^	Subset of international classification concepts for nursing practice for the care of patients with multiple myeloma	Article (2)
3	UFF^||^	2015	MP^¶^	Terminology subset CIPE^®§^ for patients in palliative care with tumor wounds	Article (2)
4	UFPB^†^	2017	D**	Validation of the CIPE^®§^ terminology subset for patients with cancer pain	Not identified
5	UFPB^†^	2009	MA^‡^	CIPE^®§^ catalog for patients with congestive heart failure	Article (2)
6	UFPB^†^	2014	D**	Validation of the CIPE^®§^ terminological subset for the elderly	Article (1)
7	UFPB^†^	2017	MA^‡^	CIPE^®§^ terminology subset proposal for elderly women with HIV^††^/AIDS^‡‡^ related vulnerability^††^	Book (1), articles (4), summaries at events (8)
8	UFPB^†^	2017	D**	Terminology subset of CIPE^®§^, structured in ontology, for the self-care of the person with stoma of intestinal elimination	Article (1), summaries at events (2)
9	UFPB^†^	2014	D**	Terminology subset of CIPE^®§^ for people with diabetes mellitus in specialized care	Articles (2), book chapter (1), event summary (1)
10	UFPB^†^	2014	MA^‡^	CIPE^®§^ nursing diagnoses/results and interventions for institutionalized elderly people	Article (1)
11	UFPB^†^	2013	MA^‡^	Proposal for a CIPE^®§^ terminology subset for clients undergoing prostatectomy	Articles (2), abstracts at events (2)
12	UFPB^†^	2011	MA^‡^	Nursing diagnoses/results and interventions for the elderly: proposal for a terminology subset of CIPE^®§^	Articles (3), book chapters (2), book (1), event summaries (5)
13	UFS^§§^	2017	D**	Terminology subset of CIPE^®§^ in traumatic brain injury	Article (2)
14	UNB^||||^	2017	D**	Terminology subset CIPE^®§^ for environmental and occupational nursing practice	Not identified
15	UFF^||^	2016	MP^¶^	Terminology subset of CIPE^®§^ for patients with Parkinson's disease in rehabilitation	Article (6)
16	UFBA^¶¶^	2014	D**	Palliative care nursing for dying with dignity: CIPE^®§^ terminology subset	Articles (2), Event summaries (2)
17	USP***	2015	D**	Construction of a CIPE^®§^ terminological subset for children and adolescents vulnerable to domestic violence	Article (1), book chapter (1), event summary (1)
18	UFES^†††^	2017	MP^¶^	Terminology subset CIPE^®§^ for the person affected by acute myocardial infarction	Article (1), summaries at events (2)
19	UFRN^‡‡‡^	2016	MA^‡^	CIPE^®§^ nursing diagnoses, results and interventions for people living with acquired immunodeficiency syndrome	Articles (5), abstract in event (1)
20	UFS^§§^	2016	MA^‡^	Terminology subset of CIPE^®§^ for infants with allergy to cow's milk protein	Event summary (1)
21	UECE^§§§^	2014	MA^‡^	CIPE^®§^ terminology subset proposal for clinical nursing practice for the elderly in primary care	Article (2)
22	PUC^||||||^	2014	MA^‡^	Terminology subset of CIPE^®§^ for nursing care in primary health care	Book chapter (1)
23	UFPB^†^	2012	MA^‡^	CIPE^®§^ terminology subset proposal for nursing practice for hypertensive patients in primary care	Articles (2), book chapter (1), event summaries (2)
24	UFF^||^	2018	MA^‡^	Terminology subset of CIPE^®§^ for patients with cancer-associated venous thromboembolism	Article (1)
25	USP***	2014	D**	Construction of a CIPE^®§^ catalog for monitoring the development of children aged 0 to 3 years	Article (1), book chapter (1), event summary (1)
26	UFG^¶¶¶^	2017	D**	Terminology subset of CIPE^®§^ for the care of people with leprosy	Book chapter (1)
27	UFES^†††^	2017	MP^¶^	Caring for the person with venous ulcer: terminology subset of CIPE^®§^	Article (1)
28	UFES^†††^	2013	MP^¶^	Care protocol for patients undergoing chemotherapy	Not identified
29	UFES^†††^	2013	MP^¶^	Nursing diagnoses and interventions for people with colostomy: a care technology	Article (2)
30	UFPB^†^	2017	MA^‡^	Validation of the nomenclature of diagnoses, results and nursing interventions for surgical clinic at UFPB University Hospital^†^	Articles (2), books (2), abstracts in event (4)
31	UNB^||||^	2013	MA^‡^	Nursing diagnoses, results and interventions for nursing practice in the context of ecological and occupational care	Not identified
32	UECE^§§§^	2014	MA^‡^	Nursing diagnoses, results and interventions in the elderly with skeletal muscle trauma of the lower limbs: foundations for the nurse's clinical practice	Event summary (1)
33	UNIFAL****	2014	MA^‡^	Nursing diagnoses, interventions and results of anxiety and fear in students of a public university	Event summary (1)

* IES = Under-graduation Institution

† UFPB = Federal University of Paraíba;

‡ MA = Academic Master's Degree;

§ International Classification for Nursing Practice;

|| UFF = Fluminense Federal University;

¶ MP = Professional Master;

** D = Doctorate;

†† HIV = Human Immunodeficiency Virus;

‡‡ AIDS = Acquired Immunodeficiency Syndrome;

§§ UFS = Federal University of Sergipe;

|||| UNB = University of Brasília;

¶¶ UFBA = Federal University of Bahia;

*** USP = University of São Paulo;

††† UFES = Federal University of Espírito Santo;

‡‡‡ UFRN = Federal University of Rio Grande do Norte;

§§§ UECE = State University of Ceará;

|||||| PUC = Pontifical Catholic University of Paraná;

¶¶¶ UFG = Federal University of Goiás;

**** UNIFAL = Universidad Federal de Alfenas

**Table 2:** Distribution of the methodological characteristics of the analyzed productions

Study	Completion	Terms validation	Cross mapping/ version	Validation of statements	Term collection
1	68 diagnosis/outcomes and 116 interventions	NI*	CIPE^®†^ Version 1.1	NI*	Bibliographical review
2	28 diagnosis and 27 interventions groups; 29 outcomes	Nurses	CIPE^®†^ version 1.1 and 2.0	Nurses (9)	Bibliographical review
3	43 diagnosis and 122 interventions	Nurses	CIPE^®†^ version 2013		Bibliographical review
4	68 diagnosis	Nurses	CIPE^®†^ version 2013	Clinical cases studies	Bibliographical review
5	68 diagnosis; 234 nursing interventions	NI*	CIPE^®†^ Version 1.1	NI*	Focus axis terms
6	105 diagnosis and 441 interventions	Nurses	CIPE^®†^2013	Nurses (5)	CIPE^®†^ version 2013
7	53 diagnosis/outcomes and 218 interventions	Nurses	CIPE^®†^ 2015	NI*	Terms Catalog
8	78 diagnosis/outcomes and 103 interventions	Nurses	CIPE^®†^ 2015	Nurses (71)	Literature and medical records review
9	66 diagnosis/outcomes and 347 interventions	Nurses (13)	CIPE^®†^ 2011	Nurses (13)	Medical records
10	60 diagnosis/outcomes and 383 interventions	Nurses	CIPE^®†^ 2013	Nurses (9)	Clinical exam
11	38 diagnosis/outcomes and 210 interventions	Nurses and Professors	CIPE^®†^ 2011	Nurses and Professors	Dissertation
12	129 diagnosis/outcomes and 627 interventions	NI*	CIPE^®†^ 2011	NI*	Mother project
13	111 diagnosis/outcomes and 144 interventions	Nurses	CIPE^®†^ 2015	Nurses (25)	Bibliographical review
14	114 diagnosis/outcomes and 219 interventions	Nurses (4)	CIPE^®†^2015	Nurses (4)	Protocols.
15	113 diagnosis/outcomes 56 interventions	Nurses (13)	CIPE^®†^ 2013	Nurses (13)	Medical records
16	33 diagnosis/outcomes and 220 interventions	Nurses	CIPE^®†^ 2011	Nurses (39)	Interview with nurses
17	139 diagnosis/outcomes and 222 interventions	NI*	CIPE^®†^ 2011	NI*	Bibliographical review
18	34 diagnosis/outcomes and 68 interventions	Nurses (22)	CIPE^®†^ 2015	Nurses (22)	Bibliographical review
19	96 diagnosis and 191 interventions	Nurses (26)	CIPE^®†^ 2013	Nurses (26)	Clinical exam
20	137 diagnosis/outcomes and 126 actions	Nurses (12)	CIPE^®†^ 2015	Nurses (12)	Medical records
21	127 diagnosis/outcomes and 515 interventions	Nurses (5)	CIPE^®†^ 2013	Nurses (5)	Official documents
22	132 diagnosis and 541 interventions	Nurses (2)	NI*	Nurses (2)	Project worksheets
23	59 diagnosis/outcomes and 351 interventions	Nurses	CIPE^®†^ 2011	Nurses	Ministry of Health Publications
24	37 nursing diagnosis and interventions	Nurses	CIPE^®†^ 2017	Nurses (34)	Bibliographical review
25	17 diagnosis and respective interventions	Nurses	CIPE^®†^ version 2.0	Nurses (51)	Bibliographical review
26	81 diagnosis/outcomes and 303 interventions	NI*	CIPE^®†^ 2015	NI*	Ministry of Health Publications/Nursing Consultations
27	86 diagnosis and 308 outcomes	Nurses (13)	CIPE^®†^ 2015	Nurses (13)	Bibliographical review
28	99 diagnosis and 175 interventions	NI*	CIPE^®†^ 2011	NI*	Bibliographical review
29	123 diagnosis and 231 interventions	NI*	CIPE^®†^ 2011	NI*	Bibliographical review
30	46 diagnosis/outcomes and 72 interventions	Nurses	CIPE^®†^ 2015	Nurses (8)	Cross-mapping
31	52 diagnosis/outcomes and 227 interventions	NI*	CIPE^®†^ 2011	NI*	Protocols.
32	97 diagnosis, 109 outcomes and 183 interventions	NI*	CIPE^®†^ version 2.0	NI*	Instrument
33	31 interventions and 1 nursing result for anxiety and another for fear	Advisor	CIPE^®†^ version 2.0	NI*	Instrument

* NI = Not identified/informed;

† CIPE = International Classification for Nursing Practice

Regarding the publicizing of results, diffusion predominated through 49 scientific articles that originated from 24 (n=72.7%) studies, followed by others that originated book chapters (n=8), books (=5) and presentation of papers at scientific events in the format of abstracts (n=37).

## Discussion

The analysis of studies on the development of CIPE^®^ terminological subsets made it possible to apprehend that the production of knowledge with a focus on the theme is unstable, as there was an increase in the production of dissertations and theses. It is believed that this reality is associated with worldwide mobilization and, mainly, with Brazil, as it is a theme that has been shown to be relevant for research and assistance.

In Brazil, the Graduate Programs in Nursing have expanded, which reflects the increase in studies permeated by the theme in Doctoral and Academic Master’s Courses and, relatively, in Professional Masters, since studies 2, 3, 15, 18, 27, 28 and 29 were developed in these courses^(^ 16 ^)^. This growth may have influenced the academic production that occurred progressively and expanded the development of the theme over the years. Thus, the training of professionals who know how to develop and use research to add value to their professional activities, with a critical analysis of work practice, stimulates the development and implementation of technological and innovation production to qualify care in the context of health^(^ 17 ^)^.

From the observed data, it appears that the publication of the first studies produced in the Brazilian *stricto sensu* graduate programs on the construction of these subsets from 2009, at UFPB, is justified by the fact it was, only in 2007, that a method to systematize its development was disclosed at an international event, which culminated in the publication of the Guide for the Development of CIPE^®^ Catalogs by the CIE the following year^(^ 12 ^)^.

The results indicated the non-regularity of production on subsets, observing peaks in 2014 and 2017 in contrast to the other years in which production ranged from 0 (2010) to 4 (2016), which differs from study published in 2014, which pointed out only 7 master’s dissertations prepared with a focus on creating subsets^(^ 12 ^)^.

This fact is justified due to the constant development of studies involving CIPE^®^, in which its versions are submitted to successive processes of revisions and updates and, their translation into Brazilian Portuguese from the Beta 2 version. Another fact that contributed to the dissemination of knowledge of this classification system was the creation of version 1.1 and, from then on, the *online* publicizing on the CIE’s *website* of the versions of the CIPE^®^.

A Polish study points out that despite the extensive promotion and training of CIE members worldwide, there are still many countries in which CIPE^®^ has not been implemented as a standard tool in health facilities. As a result, several initiatives were carried out in cooperation with local and state authorities to disseminate this Classification in health units. However, in Brazil, this reality has been shown to be different regarding the progressive aspect in the nursing teaching, research and practice scenarios^(^ 18 ^)^.

The Federal University of Paraíba Program leads the production of theses and dissertations on the development of CIPE^®^ terminological subsets in Brazil, followed by Fluminense Federal and Federal of Espírito Santo. The first stands out in relation to the number of academic productions due to the creation of the CIPE^®^ Centre in the Graduate Program in Nursing of the referred university, in 2007, which has developed the components of the life cycle of the terminology. This Brazilian accreditation made it possible, for example, to translate the CIPE^®^ into Brazil, content publications related to the theme and partnership with other states and research groups across the country^(^ 19 ^)^.

Furthermore, in order to enhance the use of CIPE^®^, the CIE has encouraged the participation of nurses, organizations and Nursing Teaching and Research Centers from around the world in the development and testing for validation of subsets terminology and its dissemination as an alternative to unify the language of nursing, as well as to identify, explain and evaluate the elements that describe its practice.

Thus, the activities of research and production of new knowledge developed in groups encourage the sectorization by areas of interest and bring together researchers and professionals*experts* interested in the theme, which encourages the theoretical deepening and the domain of practice in your field of knowledge^(^ 20 ^)^.

Regarding the non-identification of the item ‘health services’, this is because the CIE prerogatives for the construction of subsets determine that they may also be related to a nursing phenomenon, such as cancer pain, heart failure, domestic violence, palliative care, among others. *A priori*, there is no determination of a health service type scenario^(^ 12 ^)^.

The concern of a research must be sustained in the feedback that it can provide to the individuals who receive the care proposed by Nursing. In this study, this was evidenced due to the results aimed at the subjects, be they men and women, in the age groups corresponding to childhood and the elderly phase, and their peculiarities in the face of a health problem. The most common problems that justified the construction of terminological subsets were due to the character and context of specialized care for chronic non-communicable diseases and the epidemiological profile that, in this case, was evidenced by the number of studies aimed at the elderly.

The researchers’ concern with the health context experienced in Brazil is understood, and the nursing process can be used in favor of comprehensive care and be able to recognize the social and health needs of these individuals^(^ 21 ^-^ 23 ^)^.

From this point of view, the foundation that supports the research presented here are nursing theories and theoretical concepts for health. The results of this study showed a variety of views that supported them and the importance of directing the theoretical constructs in support of the nursing process, since it needs to be understood by nurses and appropriate for the reality in which the nursing service is inserted^(^ 24 ^)^.

Horta’s model, the most recurrent, brought significant epistemological support to the studies found because she was the first nurse in Brazil, which contributed to the knowledge about theory, in the professional field, to advance to guide clinical models of nursing in the country, in the perspective of describing and explaining the care realities, and to promote the constitution of theory, research and practice. From this point of view, the contribution of a theory aims to consolidate and explain the practice through concepts that express the development of actions and that explain the professionals’ world-view, that is, it is an integral part of the documentation of importance for Nursing and provides the very organization and presentation of the subset^(^ 25 ^-^ 26 ^)^.

Regarding the methodological characteristics, for the development of the subsets, 21 studies were classified as a methodological research, since they dealt with the development, validation and evaluation of tools and require demands to evaluate solid and reliable results through rigorous testing of interventions and sophisticated procedures for obtaining data^(^ 27 ^)^.

Furthermore, methodological studies require the creation of an instrument, which demand cost and time, thus implying the search for conceptual and theoretical aspects in the choice of questions, domains and items that will better explore the construct of interest^(^ 28 ^)^.

In the foreground, the search or validation of terms in the research was done, primarily, by the source of the literature review, by the evaluation of medical records, official protocols, clinical examination and interviews with professionals, who became the empirical bases. Authors point out that, regardless of how the terms are collected (manual or automated), they must be decomposed, organized and normalized, through a powerful description, by enabling the reproducibility of the method and comparisons between the results presented^(^ 9 ^,^ 29 ^)^.

In the background, there is the mapping which is done, by comparing the records of the patients’ records or literature with the standardized language, through *cross-mapping*. The results of applying this technique can help professionals to visualize terms that they use daily and that are registered in a non-standardized way^(^ 30 ^)^. In the studies found in this research, the mapping was unanimous.

A multi-center Italian study pointed to cross-mapping as an essential step in the development of a subset of nursing diagnoses in medical and surgical clinics, and the results contributed to the construction of statements in the identification of acute health responses, but also to the behavioral education of individuals and their self-care^(^ 31 ^)^.

Regarding the statements and their respective nursing diagnoses/results and interventions, a variation of 31 to 139 and 68 to 627 was found, respectively. The diversity of nursing statements presented by the specific clientele denotes the variety of care to be judged and provided by nursing professionals; thus the survey of such varied statements gives greater clinical power to the professional nurse.

The virtue of this variation is due to the diversity of techniques for collecting terms and validation that researchers use in the construction of subsets. This is a proposal encouraged by the CIE, however without providing detailed information on the method or theoretical model to be used, which causes a plurality in the standardization and organization of subsets.

Thus, in order to increase the reliability of the subsets, it is necessary to submit them to a validation process, refine the set of clinical indicators and make it reliable. Validation is a determining factor in the choice and/or application of a measurement instrument and is measured by the extent or degree to which the data represents the concept that the instrument proposes to measure^(^ 32 ^)^.

Thus, it is suggested that the group of experts or judges to validate the statements meet a set of inclusion criteria pre-established by the authors. The method details the way in which experts approach and analyze the congruence of statements, recommending the use of the content validity index (CVI) method, which measures the percentage of experts who are in agreement on certain aspects of the instrument and its items, using a *Likert* scale of 4 points^(^ 9 ^)^. There is also the inter-observer agreement index (índice *de concordância*, IC), which is performed using the formula $\mathrm{IC}=C/\left(\mathrm{NC}+C\right)$, where C is the number of agreements and NC, the number of disagreements. The terms are considered validated when they reach a CI ≥ 0.80, as this is the value considered as ideal in the literature^(^ 33 ^)^.

Content validation was used in 21 studies (63.6%), since it is a method based, necessarily, on the judgment made by individuals with experience in the content area, who have the function of analyze the items and judge whether they are comprehensive and representative or whether the content of each item relates to what you want to measure^(^ 34 ^)^. However, studies cited other validations, such as study 7, which refers to semantic validation, in which the items are understood by members of the population for which the instrument is intended, and studies 17, 20 and 30, which benefited from the validation consensus, a process through which experts review the content of a domain of knowledge related to their experience and work to reach consensus on that domain, with collective opinion or an agreement between specialists on a certain phenomenon as the best clinical practice.

However, it is clear that the criteria for selecting the judges in the content validation are made with the idealization of the authors themselves, which generates discussions and questions to determine their best profile. Studies such as 13, 15, 16, 19, 21, 24 and 25 cited the model proposed by Richard Fehring, which proposes criteria to know to what extent the experts really are specialists^(^ 35 ^)^. These criteria are given a weight and, to be considered as an expert, the sum of the scores must reach a minimum of 5 points, but this is not a feasible indication in practice.

The literature points out that there are few studies that present comments on the subject, which can make the selection of specialists a peculiar step to be fulfilled, since the author points out the favoring of academic training over clinical experience^(^ 36 ^)^. To find these specialists, we use, for example, the Lattes Platform or participants close to the main researcher, using the sample by intention or the snowball technique. Therefore, it is difficult to attract specialists, since few are willing to participate in the studies.

The inadequate choice of professionals involved in the content validation process can influence the reliability of the results and negatively impact the structuring of the subset. Therefore, it is recommended that the formation of the committee of judges to validate the terms/concepts and statements of diagnoses, results and nursing intervention should obey well-defined selection criteria, taking into account their qualification through the investigation of experience, knowledge, skill and practice of each professional involved in relation to what one wishes to validate^(^ 12 ^)^.

Another relatively used path for the conformation of the subset is clinical validation, an essential step, since it is able to refine the statements and ensure uniformity in the identification or classification process, which contributes to clarify the accuracy of this step of the process and to assist the selection of truly identifiable clinical indicators. In the results found in this research, the development of recent studies was evidenced, which points to the need to increase this aspect^(^ 37 ^)^.

For the Brazilian method, the clinical validation of the subset is a step subsequent to its development, but it presents, in the stages of identification of terms and in the construction of utterances, the content validation of terms and utterances. The terms validation process, isolated or pre-combined, is detailed^(^ 9 ^)^.

Studies 4, 14 and 30 have appropriated clinical validation, usually through nursing consultation or case studies. With this data, it can be understood that clinical validation is still a step that needs to be explored and grounded to improve the applicability of subsets in everyday clinical practice. This limitation should be treated with more care, since clinical validation research is usually linked to a single nursing diagnosis and not to a terminological subset^(^ 9 ^)^.

On the other hand, the subsets were disseminated by the authors through scientific articles and works in scientific events, mainly in the national space, in addition to chapters and books dedicated to the theme. This has enabled the dissemination and adoption of a standardized and proper system to diagnose, intervene and evaluate the consequence of the care provided to individuals, families and communities in different spaces of nursing practice. The constant use of subsets brings benefits, such as the improvement of their actions, with a reflective, effective and efficient performance, and, consequently, improves the communicative and relational process between the nurse and the other members of the health team, since it promotes recognition and visibility to the profession in different contexts and scenarios of clinical practice^(^ 12 ^)^.

When disseminating the proposal of subsets in a greater number of countries, it is expected that the adoption of a language can be particular and thus provide subsidies to diagnose, intervene and evaluate the impact of the care dedicated to the individual, family and community. The use of the CIPE^®^ subset grants the universalization of nursing language and thus recognizes, clarifies and evaluates your clinical practice, improving its actions based on reflective and effective judgment, assisting the relational and communication process between health team members. Thus, it favors recognition and makes the performance of nursing visible in the context of their daily practices^(^ 38 ^)^.

Its applicability and structuring depend on the involvement of the professional in nursing practice and on the processes that are apprehended, in a unique way, through relationships, various influences, the state of physical and emotional security, the environment, as well as individual potentialities, of beliefs, values and cultural aspects^(^ 12 ^,^ 39 ^)^.

In addition, these subsets can facilitate the documentation workload among nurses and facilitate the storage and retrieval of standardized data for various purposes, such as quality improvement, management decision support and research. Health documentation makes it possible to provide data that can be communicated, compared and evaluated more fluently for various health professionals and clinical environments^(^ 40 ^)^.

Regarding the limitations of this research, we can mention the fact that there are few studies added when applying the inclusion criteria. Of the total 124, only 34 established subsets. This demonstrates that the discussion about the CIPE^®^ has been inserted in several aspects, as in investigations that were reduced to finding terms, performing the mapping or building the diagnostic statements. Another limitation involved the search for studies, as the dissemination of the complete work is still an arduous path for reading in full for the detailed search of the steps used.

Finally, the study promotes advances in the knowledge of nursing science by demonstrating the increased use of CIPE^®^ as a standardized language system for the terminological organization of nursing care and which is configured as a resource of unquestionable relevance for carrying out systematic assistance. Furthermore, it points out that its use may favor clinical practice and clinical reasoning, supporting safe and planned communication, with visibility for actions in teaching, research, management and assistance.

As suggestions, it is recommended to promote discussions regarding the validations to improve the result of the subsets, since three studies contemplated clinical validation, which resumes the need to continue research and to find better strategies. This is a way of valuing nursing as a science, as it unifies its language and contributes to improving communication and the quality of care that is given to people.

## Conclusion

Based on the paths of the published literature that presents the CIPE^®^, it appears that subsets are being developed in Brazil for various populations and environments with a view to ensuring the universalization of the nursing language. However, the use of this system in generating new knowledge and validating the practice is still progressing, giving the opportunity to use well-developed systems in their current state to deepen what is known about nursing practice and how to better demonstrate advances in nursing. patient outcomes through nursing care.

On the other hand, the studies pointed out weaknesses regarding the standardization of the stages of elaboration of the subsets, the detailing of the descriptions of some methodological steps and the content and clinical validation of the identified terms and concepts that can directly interfere with the reliability of the final product of these surveys.
